# Supplementary material for: Complex pattern of facial remapping in somatosensory cortex following congenital but not acquired hand loss
Source: eLife. 2022 Dec 30;11:e76158. doi: 10.7554/eLife.76158 (PMC9851617; doi:10.7554/eLife.76158)
Supplement: Figure 2—source data 1. [file elife-76158-fig2-data1.docx]

| **Within Subjects Effects** | | | | | | | | | | | | | |
| --- | --- | --- | --- | --- | --- | --- | --- | --- | --- | --- | --- | --- | --- |
| **Cases** | | **Sum of Squares** | | **df** | | **Mean Square** | | **F** | | **p** | | **η²_p_** | |
| Hemisphere |  | 2.610 |  | 1 |  | 2.610 |  | 0.164 |  | 0.688 |  | 0.005 |  |
| Hemisphere ✻ group |  | 0.308 |  | 1 |  | 0.308 |  | 0.019 |  | 0.890 |  | 0.001 |  |
| Hemisphere ✻ brainVol |  | 3.401 |  | 1 |  | 3.401 |  | 0.214 |  | 0.646 |  | 0.006 |  |
| Residuals |  | 571.476 |  | 36 |  | 15.874 |  |  |  |  |  |  |  |
|  | | | | | | | | | | | | | |
| \| **Between Subjects Effects** \| \| \| \| \| \| \| \| \| \| \| \| \| \| \| \| --- \| --- \| --- \| --- \| --- \| --- \| --- \| --- \| --- \| --- \| --- \| --- \| --- \| --- \| --- \| \| **Cases** \| \| **Sum of Squares** \| \| **df** \| \| **Mean Square** \| \| **F** \| \| **p** \| **η²_p_** \| \| \| \| \| Group \|  \| 92.0 \|  \| 1 \|  \| 92.0 \|  \| 2.25 \|  \| 0.143 \|  \| 0.059 \|  \| \| BrainVol \|  \| 137.3 \|  \| 1 \|  \| 137.3 \|  \| 3.35 \|  \| 0.075 \|  \| 0.085 \|  \| \| Residuals \|  \| 1474.9 \|  \| 36 \|  \| 41.0 \|  \|  \|  \|  \|  \|  \|  \| \|  \| \| \| \| \| \| \| \| \| \| \| \| \| \| \| \| *Note.*  Type III Sum of Squares \| \| \| \| \| \| \| \| \| \| \| \| \| \| \| | | | | | | | | | | | | | |

***Figure 2 – source data 1. Main effects and interaction for comparison of geodesic distances between amputees and controls for the lips.***
