# Supplementary material for: Complex pattern of facial remapping in somatosensory cortex following congenital but not acquired hand loss
Source: eLife. 2022 Dec 30;11:e76158. doi: 10.7554/eLife.76158 (PMC9851617; doi:10.7554/eLife.76158)
Supplement: Figure 2—source data 2. [file elife-76158-fig2-data2.docx]

| **Within Subjects Effects** | | | | | | | | | | | | | |
| --- | --- | --- | --- | --- | --- | --- | --- | --- | --- | --- | --- | --- | --- |
| **Cases** | | **Sum of Squares** | | **df** | | **Mean Square** | | **F** | | **p** | | **η²_p_** | |
| Hemisphere |  | 5.57 |  | 1 |  | 5.57 |  | 0.312 |  | 0.579 |  | 0.008 |  |
| Hemisphere ✻ group |  | 77.67 |  | 1 |  | 77.67 |  | 4.352 |  | 0.043 |  | 0.098 |  |
| Hemisphere ✻ brainVol |  | 1.43 |  | 1 |  | 1.43 |  | 0.080 |  | 0.778 |  | 0.002 |  |
| Residuals |  | 713.93 |  | 40 |  | 17.85 |  |  |  |  |  |  |  |
|  | | | | | | | | | | | | | |
| \| **Between Subjects Effects** \| \| \| \| \| \| \| \| \| \| \| \| \| \| \| --- \| --- \| --- \| --- \| --- \| --- \| --- \| --- \| --- \| --- \| --- \| --- \| --- \| --- \| \| **Cases** \| \| **Sum of Squares** \| \| **df** \| \| **Mean Square** \| \| **F** \| \| **p** \| \| **η²_p_** \| \| \| Group \|  \| 3.78 \|  \| 1 \|  \| 3.78 \|  \| 0.084 \|  \| 0.774 \|  \| 0.002 \|  \| \| BrainVol \|  \| 483.27 \|  \| 1 \|  \| 483.27 \|  \| 10.737 \|  \| 0.002 \|  \| 0.212 \|  \| \| Residuals \|  \| 1800.44 \|  \| 40 \|  \| 45.01 \|  \|  \|  \|  \|  \|  \|  \| \|  \| \| \| \| \| \| \| \| \| \| \| \| \| \| \| *Note.*  Type III Sum of Squares \| \| \| \| \| \| \| \| \| \| \| \| \| \| | | | | | | | | | | | | | |

***Figure 2 – source data 2. Main effects and interaction for comparison of geodesic distances of the lips between one-handers and controls.***
