# Supplementary material for: Complex pattern of facial remapping in somatosensory cortex following congenital but not acquired hand loss
Source: eLife. 2022 Dec 30;11:e76158. doi: 10.7554/eLife.76158 (PMC9851617; doi:10.7554/eLife.76158)
Supplement: Figure 3—source data 1. [file elife-76158-fig3-data1.docx]

| **Within Subjects Effects** | | | | | | | | | | | | | |
| --- | --- | --- | --- | --- | --- | --- | --- | --- | --- | --- | --- | --- | --- |
| **Cases** | | **Sum of Squares** | | **df** | | **Mean Square** | | **F** | | **p** | | **η²_p_** | |
| Hemisphere |  | 6.39 |  | 1 |  | 6.39 |  | 0.348 |  | 0.559 |  | 0.010 |  |
| Hemisphere ✻ group |  | 24.57 |  | 1 |  | 24.57 |  | 1.338 |  | 0.255 |  | 0.036 |  |
| Hemisphere ✻ brainVol |  | 4.77 |  | 1 |  | 4.77 |  | 0.260 |  | 0.613 |  | 0.007 |  |
| Residuals |  | 660.72 |  | 36 |  | 18.35 |  |  |  |  |  |  |  |
|  | | | | | | | | | | | | | |
| \| **Between Subjects Effects** \| \| \| \| \| \| \| \| \| \| \| \| \| \| \| --- \| --- \| --- \| --- \| --- \| --- \| --- \| --- \| --- \| --- \| --- \| --- \| --- \| --- \| \| **Cases** \| \| **Sum of Squares** \| \| **df** \| \| **Mean Square** \| \| **F** \| \| **p** \| \| **η²_p_** \| \| \| Group \|  \| 12.4 \|  \| 1 \|  \| 12.4 \|  \| 0.470 \|  \| 0.497 \|  \| 0.013 \|  \| \| BrainVol \|  \| 69.6 \|  \| 1 \|  \| 69.6 \|  \| 2.636 \|  \| 0.113 \|  \| 0.068 \|  \| \| Residuals \|  \| 951.2 \|  \| 36 \|  \| 26.4 \|  \|  \|  \|  \|  \|  \|  \| \|  \| \| \| \| \| \| \| \| \| \| \| \| \| \| \| *Note.*  Type III Sum of Squares \| \| \| \| \| \| \| \| \| \| \| \| \| \| | | | | | | | | | | | | | |

***Figure 3 – source data 1. Main effects and interaction for comparison of geodesic distances between amputees and controls for the forehead.***
