# Supplementary material for: Complex pattern of facial remapping in somatosensory cortex following congenital but not acquired hand loss
Source: eLife. 2022 Dec 30;11:e76158. doi: 10.7554/eLife.76158 (PMC9851617; doi:10.7554/eLife.76158)
Supplement: Figure 3—source data 2. [file elife-76158-fig3-data2.docx]

| **Within Subjects Effects** | | | | | | | | | | | | | |
| --- | --- | --- | --- | --- | --- | --- | --- | --- | --- | --- | --- | --- | --- |
| **Cases** | | **Sum of Squares** | | **df** | | **Mean Square** | | **F** | | **p** | | **η²_p_** | |
| Hemisphere |  | 0.621 |  | 1 |  | 0.621 |  | 0.041 |  | 0.841 |  | 0.001 |  |
| Hemisphere ✻ group |  | 113.146 |  | 1 |  | 113.146 |  | 7.437 |  | 0.009 |  | 0.157 |  |
| Hemisphere ✻ brainVol |  | 0.796 |  | 1 |  | 0.796 |  | 0.052 |  | 0.820 |  | 0.001 |  |
| Residuals |  | 608.545 |  | 40 |  | 15.214 |  |  |  |  |  |  |  |
|  | | | | | | | | | | | | | |
| \| **Between Subjects Effects** \| \| \| \| \| \| \| \| \| \| \| \| \| \| \| --- \| --- \| --- \| --- \| --- \| --- \| --- \| --- \| --- \| --- \| --- \| --- \| --- \| --- \| \| **Cases** \| \| **Sum of Squares** \| \| **df** \| \| **Mean Square** \| \| **F** \| \| **p** \| **η²_p_** \| \| \| \| Group \|  \| 108.8 \|  \| 1 \|  \| 108.8 \|  \| 3.257 \|  \| 0.079 \|  \| 0.075 \|  \| \| \| BrainVol \|  \| 31.4 \|  \| 1 \|  \| 31.4 \|  \| 0.939 \|  \| 0.338 \|  \| 0.023 \|  \| \| \| Residuals \|  \| 1336.0 \|  \| 40 \|  \| 33.4 \|  \|  \|  \|  \|  \|  \|  \| \| \|  \| \| \| \| \| \| \| \| \| \| \| \| \| \| \| *Note.*  Type III Sum of Squares \| \| \| \| \| \| \| \| \| \| \| \| \| \| | | | | | | | | | | | | | |

***Figure 3 – source data 2. Main effects and interaction for comparison of geodesic distances between one-handers and controls for the forehead.***
