# Supplementary material for: Complex pattern of facial remapping in somatosensory cortex following congenital but not acquired hand loss
Source: eLife. 2022 Dec 30;11:e76158. doi: 10.7554/eLife.76158 (PMC9851617; doi:10.7554/eLife.76158)
Supplement: Figure 4—source data 1. [file elife-76158-fig4-data1.docx]

| **Within Subjects Effects** | | | | | | | | | | | | | |
| --- | --- | --- | --- | --- | --- | --- | --- | --- | --- | --- | --- | --- | --- |
| **Cases** | | **Sum of Squares** | | **df** | | **Mean Square** | | **F** | | **p** | | **η²_p_** | |
| Hemisphere |  | 3.93 |  | 1 |  | 3.93 |  | 0.328 |  | 0.570 |  | 0.009 |  |
| Hemisphere ✻ group |  | 58.29 |  | 1 |  | 58.29 |  | 4.859 |  | 0.034 |  | 0.119 |  |
| Hemisphere ✻ brainVol |  | 6.59 |  | 1 |  | 6.59 |  | 0.549 |  | 0.463 |  | 0.015 |  |
| Residuals |  | 431.83 |  | 36 |  | 12.00 |  |  |  |  |  |  |  |
|  | | | | | | | | | | | | | |
| \| **Between Subjects Effects** \| \| \| \| \| \| \| \| \| \| \| \| \| \| \| --- \| --- \| --- \| --- \| --- \| --- \| --- \| --- \| --- \| --- \| --- \| --- \| --- \| --- \| \| **Cases** \| \| **Sum of Squares** \| \| **df** \| \| **Mean Square** \| \| **F** \| \| **p** \| \| **η²_p_** \| \| \| Group \|  \| 115 \|  \| 1 \|  \| 115 \|  \| 0.963 \|  \| 0.333 \|  \| 0.026 \|  \| \| BrainVol \|  \| 383 \|  \| 1 \|  \| 383 \|  \| 3.198 \|  \| 0.082 \|  \| 0.082 \|  \| \| Residuals \|  \| 4313 \|  \| 36 \|  \| 120 \|  \|  \|  \|  \|  \|  \|  \| \|  \| \| \| \| \| \| \| \| \| \| \| \| \| \| \| *Note.*  Type III Sum of Squares \| \| \| \| \| \| \| \| \| \| \| \| \| \| | | | | | | | | | | | | | |

***Figure 4 – source data 1. Main effects and interaction for comparison of geodesic distances between amputees and controls for the tongue.***
