# Supplementary material for: Complex pattern of facial remapping in somatosensory cortex following congenital but not acquired hand loss
Source: eLife. 2022 Dec 30;11:e76158. doi: 10.7554/eLife.76158 (PMC9851617; doi:10.7554/eLife.76158)
Supplement: Figure 4—source data 2. [file elife-76158-fig4-data2.docx]

| **Within Subjects Effects** | | | | | | | | | | | | | |
| --- | --- | --- | --- | --- | --- | --- | --- | --- | --- | --- | --- | --- | --- |
| **Cases** | | **Sum of Squares** | | **df** | | **Mean Square** | | **F** | | **p** | | **η²_p_** | |
| Hemisphere |  | 22.3 |  | 1 |  | 22.3 |  | 0.855 |  | 0.2361 |  | 0.021 |  |
| Hemisphere ✻ group |  | 222.9 |  | 1 |  | 222.9 |  | 8.536 |  | 0.006 |  | 0.176 |  |
| Hemisphere ✻ brainVol |  | 11.9 |  | 1 |  | 11.9 |  | 0.9455 |  | 0.504 |  | 0.011 |  |
| Residuals |  | 1044.7 |  | 40 |  | 26.1 |  |  |  |  |  |  |  |
|  | | | | | | | | | | | | | |
| \| **Between Subjects Effects** \| \| \| \| \| \| \| \| \| \| \| \| \| \| \| --- \| --- \| --- \| --- \| --- \| --- \| --- \| --- \| --- \| --- \| --- \| --- \| --- \| --- \| \| **Cases** \| \| **Sum of Squares** \| \| **df** \| \| **Mean Square** \| \| **F** \| \| **p** \| **η²_p_** \| \| \| \| Group \|  \| 225 \|  \| 1 \|  \| 225 \|  \| 2.20 \|  \| 0.146 \|  \| 0.052 \|  \| \| \| BrainVol \|  \| 331 \|  \| 1 \|  \| 331 \|  \| 3.23 \|  \| 0.080 \|  \| 0.075 \|  \| \| \| Residuals \|  \| 4090 \|  \| 40 \|  \| 102 \|  \|  \|  \|  \|  \|  \|  \| \| \|  \| \| \| \| \| \| \| \| \| \| \| \| \| \| \| *Note.*  Type III Sum of Squares \| \| \| \| \| \| \| \| \| \| \| \| \| \| | | | | | | | | | | | | | |

***Figure 4 – source data 2. Main effects and interaction for comparison of geodesic distances between one-handers and controls for the tongue.***
