# Supplementary material for: Complex pattern of facial remapping in somatosensory cortex following congenital but not acquired hand loss
Source: eLife. 2022 Dec 30;11:e76158. doi: 10.7554/eLife.76158 (PMC9851617; doi:10.7554/eLife.76158)
Supplement: Figure 5—source data 1. [file elife-76158-fig5-data1.docx]

| Fixed Effect Omnibus tests | | | | | | | | | |
| --- | --- | --- | --- | --- | --- | --- | --- | --- | --- |
|  |  |  |  |  |  |  |  |  |  |
|  | | **F** | | **Num df** | | **Den df** | | **p** | |
| Group |  | 7.45 |  | 1 |  | 240.0 |  | 0.007 |  |
| FaceParts |  | 761.17 |  | 3 |  | 240.0 |  | < .001 |  |
| Hemisphere |  | 2.12 |  | 1 |  | 240.0 |  | 0.147 |  |
| Age |  | 1.67 |  | 1 |  | 15.0 |  | 0.216 |  |
| Group ✻ FaceParts |  | 1.61 |  | 3 |  | 240.0 |  | 0.188 |  |
| Group ✻ Hemisphere |  | 7.70 |  | 1 |  | 240.0 |  | 0.006 |  |
| Hemisphere ✻ FaceParts |  | 5.52 |  | 3 |  | 240.0 |  | 0.001 |  |
| Group ✻ Hemisphere ✻ FaceParts |  | 2.39 |  | 3 |  | 240.0 |  | 0.070 |  |
| Note. Satterthwaite method for degrees of freedom | | | | | | | | | |

***Figure 5 – source data 1. Results from the linear mixed model comparing Jaccard similarity values of amputees’ maps relative to the ones of one-handers and controls respectively.***
