# Supplementary material for: Complex pattern of facial remapping in somatosensory cortex following congenital but not acquired hand loss
Source: eLife. 2022 Dec 30;11:e76158. doi: 10.7554/eLife.76158 (PMC9851617; doi:10.7554/eLife.76158)
Supplement: Figure 7—source data 1. [file elife-76158-fig7-data1.docx]

| Fixed Effect Omnibus tests | | | | | | | | | |
| --- | --- | --- | --- | --- | --- | --- | --- | --- | --- |
|  |  |  |  |  |  |  |  |  |  |
|  | | **F** | | **Num df** | | **Den df** | | **p** | |
| Group |  | 1.010 |  | 2 |  | 56.0 |  | 0.371 |  |
| Hemisphere |  | 1.301 |  | 1 |  | 627.0 |  | 0.254 |  |
| FacePairs |  | 268.322 |  | 5 |  | 627.0 |  | < .001 |  |
| Age |  | 0.282 |  | 1 |  | 56.0 |  | 0.598 |  |
| Group ✻ Hemisphere |  | 0.626 |  | 2 |  | 627.0 |  | 0.535 |  |
| Group ✻ FacePairs |  | 1.462 |  | 10 |  | 627.0 |  | 0.150 |  |
| Hemisphere ✻ FacePairs |  | 0.222 |  | 5 |  | 627.0 |  | 0.953 |  |
| Group ✻ Hemisphere ✻ FacePairs |  | 0.136 |  | 10 |  | 627.0 |  | 0.999 |  |
| Note. Satterthwaite method for degrees of freedom | | | | | | | | | |
|  | | | | | | | | | |

***Figure 7 – source data 1. Results from the linear mixed model used to explore differences in face-face pairwise distances in the face ROI for amputees, one-handers and controls.***
