# Supplementary material for: Complex pattern of facial remapping in somatosensory cortex following congenital but not acquired hand loss
Source: eLife. 2022 Dec 30;11:e76158. doi: 10.7554/eLife.76158 (PMC9851617; doi:10.7554/eLife.76158)
Supplement: Figure 7—source data 2. [file elife-76158-fig7-data2.docx]

| Fixed Effect Omnibus tests | | | | | | | | | |
| --- | --- | --- | --- | --- | --- | --- | --- | --- | --- |
|  |  |  |  |  |  |  |  |  |  |
|  | | **F** | | **Num df** | | **Den df** | | **p** | |
| Group |  | 0.7675 |  | 2 |  | 56.0 |  | 0.469 |  |
| Hemisphere |  | 0.1776 |  | 1 |  | 627.0 |  | 0.674 |  |
| Face-Face |  | 261.1649 |  | 5 |  | 627.0 |  | < .001 |  |
| Age |  | 0.0303 |  | 1 |  | 56.0 |  | 0.862 |  |
| Group ✻ Hemisphere |  | 4.3429 |  | 2 |  | 627.0 |  | 0.013 |  |
| Group ✻ Face-Face |  | 1.9339 |  | 10 |  | 627.0 |  | 0.038 |  |
| Hemisphere ✻ Face-Face |  | 0.4519 |  | 5 |  | 627.0 |  | 0.812 |  |
| Group ✻ Hemisphere ✻ Face-Face |  | 0.2969 |  | 10 |  | 627.0 |  | 0.982 |  |
| Note. Satterthwaite method for degrees of freedom | | | | | | | | | |
|  | | | | | | | | | |

***Figure 7 – source data 2. Results from the linear mixed model used to explore differences in face-face pairwise distances in the M1 face ROI for amputees, one-handers and controls.***
