# Supplementary material for: Complex pattern of facial remapping in somatosensory cortex following congenital but not acquired hand loss
Source: eLife. 2022 Dec 30;11:e76158. doi: 10.7554/eLife.76158 (PMC9851617; doi:10.7554/eLife.76158)
Supplement: Transparent reporting form [file elife-76158-transrepform1.docx]

***eLife’s* transparent reporting form**

We encourage authors to provide detailed information *within their submission* to facilitate the interpretation and replication of experiments. Authors can upload supporting documentation to indicate the use of appropriate reporting guidelines for health-related research (see [EQUATOR Network](http://www.equator-network.org/%20)), life science research (see the [BioSharing Information Resource](https://biosharing.org/)), or the [ARRIVE guidelines](http://www.plosbiology.org/article/info:doi/10.1371/journal.pbio.1000412) for reporting work involving animal research. Where applicable, authors should refer to any relevant reporting standards documents in this form.

If you have any questions, please consult our Journal Policies and/or contact us: [editorial@elifesciences.org](mailto:editorial@elifesciences.org).

**Sample-size estimation**

- You should state whether an appropriate sample size was computed when the study was being designed
- You should state the statistical method of sample size computation and any required assumptions
- If no explicit power analysis was used, you should describe how you decided what sample (replicate) size (number) to use

Please outline where this information can be found within the submission (e.g., sections or figure legends), or explain why this information doesn’t apply to your submission:

The majority of amputee research exploring cortical plasticity and phantom limb pain demonstrate a similar, if not smaller, sample size of amputees (Lotze et al., 2001). Our study, therefore, should be able to identify differences in cortical mapping between amputees with and without phantom pain, if it exists. Other work addressing cortical plasticity in one-handers and topography in controls also record similar sample sizes (Hahamy et al., 2017; Servos et al., 199).

Justification for the chosen sample size can be found in the *Discussion* (p.25) section of the manuscript.

**Replicates**

- You should report how often each experiment was performed
- You should include a definition of biological versus technical replication
- The data obtained should be provided and sufficient information should be provided to indicate the number of independent biological and/or technical replicates
- If you encountered any outliers, you should describe how these were handled
- Criteria for exclusion/inclusion of data should be clearly stated
- High-throughput sequence data should be uploaded before submission, with a private link for reviewers provided (these are available from both GEO and ArrayExpress)

Please outline where this information can be found within the submission (e.g., sections or figure legends), or explain why this information doesn’t apply to your submission:

Information relating to replications, outlier definition and exclusion/inclusion criteria of the data can be found in the *Materials and Methods* and *Supplementary Methods* section of the manuscript.

The study involved a functional magnetic resonance imaging paradigm, whereby each experimental condition was repeated 4 times per run (5 times for baseline), over 3 functional runs. This method of replication is widely accepted in the community and required for good signal-noise ratio and for multivariate analyses. Furthermore, two control participants replicated the design ~12 months apart, as well as a passive version of each experimental condition, in order to assess validity and reliability of the chosen design.

Outliers were classified as +/- 3 standard deviations to the mean. We chose to not remove outliers in our analyses and checked that the significance and direction of the results did not change if identified outliers were removed. Furthermore, motion outliers (> 0.9 mm) of large movements between imaging volumes were included as additional regressors of no interest at the individual level.

**Statistical reporting**

- Statistical analysis methods should be described and justified
- Raw data should be presented in figures whenever informative to do so (typically when N per group is less than 10)
- For each experiment, you should identify the statistical tests used, exact values of N, definitions of center, methods of multiple test correction, and dispersion and precision measures (e.g., mean, median, SD, SEM, confidence intervals; and, for the major substantive results, a measure of effect size (e.g., Pearson's r, Cohen's d)
- Report exact p-values wherever possible alongside the summary statistics and 95% confidence intervals. These should be reported for all key questions and not only when the p-value is less than 0.05.

Please outline where this information can be found within the submission (e.g., sections or figure legends), or explain why this information doesn’t apply to your submission:

Information relating to statistical reporting can be found within *Materials and Methods* and *Results* section.

Statistical analysis methods are described and justified in *Materials and Methods*. Raw data presented in figures will be attached as a source data figure supplement, especially when the figure itself includes histograms. All results of statistical analysis reported in the *Results* section included: test value, degrees of freedom, exact p-values, effect size estimate, and for non-significant results, Bayes values. Means and standard error of the means of such statistical tests can either be found in the associated figure or the text. Further information regarding key statistical tests, e.g., main effects, are included as figure supplements. Exact values of N can be found in the *Materials and Methods* section.

(For large datasets, or papers with a very large number of statistical tests, you may upload a single table file with tests, Ns, etc., with reference to sections in the manuscript.)

**Group allocation**

- Indicate how samples were allocated into experimental groups (in the case of clinical studies, please specify allocation to treatment method); if randomization was used, please also state if restricted randomization was applied
- Indicate if masking was used during group allocation, data collection and/or data analysis

Please outline where this information can be found within the submission (e.g., sections or figure legends), or explain why this information doesn’t apply to your submission:

No masking was used during group allocation, data collection and/or data analysis. Information regarding experimental groups can be found in the *Material and Methods* section.

**Additional data files (“source data”)**

- We encourage you to upload relevant additional data files, such as numerical data that are represented as a graph in a figure, or as a summary table
- Where provided, these should be in the most useful format, and they can be uploaded as “Source data” files linked to a main figure or table
- Include model definition files including the full list of parameters used
- Include code used for data analysis (e.g., R, MatLab)
- Avoid stating that data files are “available upon request”

Please indicate the figures or tables for which source data files have been provided:

Source data of all figures and tests are included in the submission.

The data generated and analysed during this study is available to the public on Open Science Framework (<https://osf.io/xq3am/>).
